# Creating transformational learning experiences for 21^st^ century healthcare students through preclinical skills training at a South African university

**DOI:** 10.1186/s12909-024-05177-9

**Published:** 2024-02-27

**Authors:** Anke van der Merwe, Corlia Janse van Vuuren

**Affiliations:** School of Health and Rehabilitation Sciences, Faculty of Health Sciences, University of the Free State, 205 Nelson Mandela Drive, Park West, 9301 Bloemfontein, Free State South Africa

**Keywords:** Graduate attributes, Preclinical skills, Healthcare education, Interprofessional education, Curriculum, Conceptual framework

## Abstract

**Background:**

Creating an inclusive interprofessional teaching and learning community can enhance student engagement and ultimately develop essential graduate attributes (GA) (also known as generic, transferable, core, soft, work-ready or nontechnical skills). The early practical development of GA within a diverse space is essential in health profession education, as students experience the transition to clinical training as challenging.

**Aim:**

This paper describes the conceptualization and implementation of an inclusive interprofessional curriculum focused on GA development in the preclinical years.

**Methods:**

A phased multimethod research design was applied. Phase 1 focused on the conceptualization of a preclinical GA development curriculum through a consensus-seeking process among all staff in the School of Health and Rehabilitation Sciences (*N* = 36). Subsequently, in Phase 2, quantitative and qualitative data were gathered from participating first-year students (*N* = 135) as an early curricular implementation review. Descriptive statistical analyses for quantitative and thematic analyses for qualitative data were performed.

**Results:**

During Phase 1, five themes were identified (Ethics, Professionalism, General principles for interventions, Organizations and institutions, Management) informing preclinical curriculum development. Forty-one first-year students (30%) participated in Phase 2. The majority of participants (87%) indicated that they had a positive learning experience during Phase 2. Students expressed that engagement was encouraged (83%) within a space of mutual respect (83%), with interprofessional groups assisting in building “*a trusting environment and a supportive one*”. Students indicated they “*liked that it [module] wasn’t just about one topic*”, as it concretized that “*there is more to being a healthcare professional that just treating people*”.

**Conclusion:**

GA development provides an invaluable opportunity for interprofessional engagement. Creating a diverse and inclusive curricular space through multimodal and interprofessional training, GA training was transformed to be more practical and future-focused, creating a positive learning experience. Future research should focus on the longer-term impact of this practical, preclinical GA development during the transition of these students into the clinical training space.

## Introduction

Higher education in its totality is instrumental in shaping the thoughts, abilities, and personalities of the future of healthcare professionals, [1] as quality education is critical in achieving national and sustainable development goals and transforming society by addressing the identified disconnect between the current higher education landscape and the rapidly changing reality [2, 3]. An important driving factor of quality assurance frameworks guiding higher education programmes is to develop responsible citizenship by ensuring that the provided educational experiences appropriately prepare graduates not only within their immediate society but also on a national, continental, and global level [2]. Curriculum renewal is thus highlighted as a specific focus area to aid learning and teaching through improved student engagement, consideration of technological advancements and to address topical issues such as for students to develop not only on a personal level but also have a constructive impact on societal transformation [2, 3].

Globally, graduates are expected to be well-rounded, highly skilled professionals who can contribute in a constructive way toward socioeconomic development, technological innovations and problem solving within a variety of environments [2, 4, 5]. Healthcare educators thus have the responsibility to prepare healthcare professionals for a future that is complex and uncertain, thereby necessitating the need to look beyond theory toward developing attributes that are personally, professionally, and socially valuable [2, 3]. Subsequently, the fluid nature of the workplace has resulted in undergraduate training institutions being mandated to increasingly focus on the development of generic interpersonal skills and abilities, popularized as graduate attributes (GA), such as communication, teamwork, complex problem-solving skills, critical thinking, and emotional intelligence [6–8]. Careful consideration should be given that developing these attributes is a process and is enabled through practice and familiarity [8, 9] aiming to develop a healthcare graduate who could be a productive contributor through enhancing social cohesion [7], allowing self-evolution and the ability to adapt to an ever-changing environment [10].

The treacherous curriculum development journey is littered with a myriad of competing priorities both explicitly and implicitly. These priorities include expectations from industry, regulatory bodies, institutional guidelines and student voice [5, 11]. A dedicated focus on creating an inclusive community during both the curriculum development and implementation process is essential to ensure that all areas are explored within an environment where unconscious bias is limited [5]. When conceptualizing the curriculum, it is paramount to balance the aforementioned external priorities with the curriculum’s commitment to diversity and inclusion, social justice and student well-being with careful consideration and knowledge of the student population that will engage in the learning experiences [11].

**Fig. 1:**
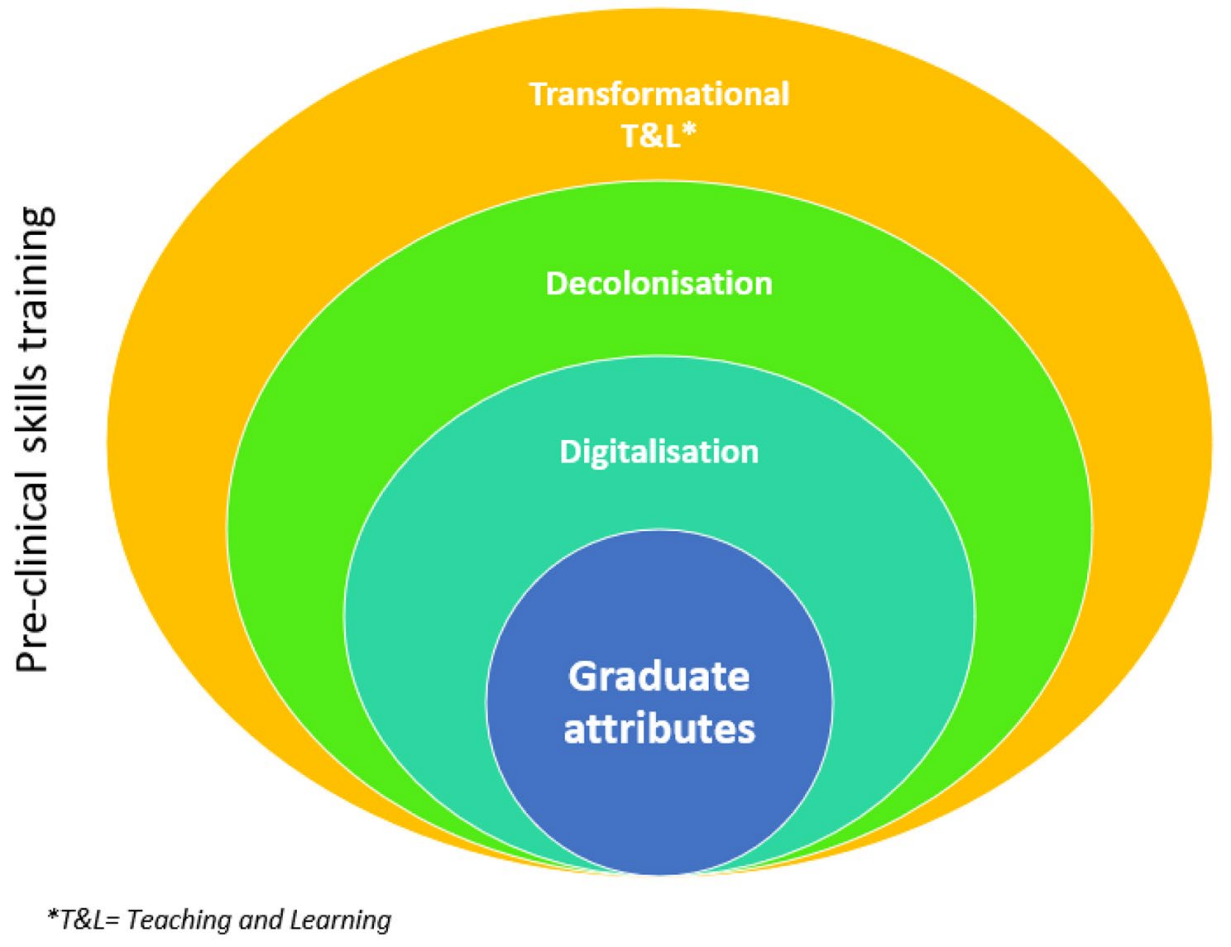
Conceptual framework underpinning the preclinical skills training curriculum development

In line with the aforementioned developments within the higher education sphere, an institutional drive for the intentional mapping and integration of GA, together with the creation of an inclusive and socially just university culture [12, 13], catapulted the need for a renewed educational approach to preclinical GA training within the School of Health and Rehabilitation Sciences (SoHRS) at the University of the Free State (UFS) to the forefront. Underpinning the continued curriculum development and/or renewal discussions within the SoHRS, comprising five departments (see Table 1) are the GA expected by the Health Professions Council of South Africa as well as institutional strategies and transformation agendas [12, 13]. These underpinnings resultantly also informed the development of the conceptual framework (see Fig. 1) for the preclinical skills training curriculum reported in this article.

**Table 1:** Departments situated within the SoHRS at the UFS.

Department	Students within department
Exercise and Sport Sciences (*n* = 8)	Biokineticits; Sport Coaching (not health science related)
Nutrition and Dietetics (*n* = 7)	Dieticians
Occupational Therapy (*n* = 7)	Occupational therapists
Optometry (*n* = 5)	Optometrists
Physiotherapy (*n* = 9)	Physiotherapists

### Conceptual framework for the proposed preclinical skills training curriculum

GA development is illustrated as the core of the proposed preclinical skills training curriculum (see Fig. 1). The factors and principles facilitating GA development are not viewed as being isolated and lone standing but rather interwoven and overlap extensively. Thus, a fluid and mindful approach to the development of the curriculum is advised, with multistakeholder input to ensure transparency and that all voices are heard during the curriculum development processes.

The early development of GA within healthcare education, including the decolonization of higher education, has received increased attention [5, 8, 10]. However, although the literature has presented interventions to develop individual GA, research guiding the full curricular integration of GA training in healthcare education remains elusive [10]. Additionally, the limited impact of decolonization interventions [5] also challenges true GA training integration in existing undergraduate healthcare programmes [5] and has resulted in a mostly ad hoc approach to decolonization, often reliant on academics interested in the topic [7].

As illustrated in the conceptual framework (see Fig. 1), not only is a consideration of decolonization essential when reviewing GA development but also societal digitalization. The emergence of a new generation of attributes, including but not limited to, demonstrating the ability to engage with technology and to develop international capabilities [7, 14], has drawn attention to the integration of digitalization into higher education. Recent literature has alluded to the fact that although undergraduate students may be technologically active, they remain digital natives [15]. Students might thus not be as well-versed in utilizing the available technology within a teaching and learning environment [16], resulting in limited healthcare professional training specifically in terms of the utilization of digital health [17]. In the best interest of health profession education, the next generation of healthcare professionals should be prepared to function effectively in a landscape incorporating technology [16] and be adept in the assessment of the relevance of technology for use in each individual situation [16, 18].

Additional barriers to the integration of GA within undergraduate healthcare education, directly linked to challenges surrounding decolonization and digitalization, are administrative, such as not addressing contextual needs, a lack of faculty development [19], and limited curriculum time for the development of these skills [8]. It is advised that training should move beyond mere content addition or removal but toward the student experience and may include aspects related to how content is taught and delivered within a digitalized environment [5, 15]. All content, including digital content, should, however, remain culturally, contextually, and technologically relevant [15, 20], inclusive of developments based in this instance on the African context [15]. It is thus vital for curriculum developers to question the underlying narratives, hierarchies and power dynamics [5] and create a shared narrative between staff and students to foster a sense of belonging [5] in creating a transformative learning environment to challenge both students and staff to move away from dualistic thinking [21].

### Research context and problem statement

Work-integrated learning, as experienced in the transition from the preclinical to the clinical environment, seems particularly challenging for students due to a lack of strategies to deal with day-to-day challenges in the clinical environment [22, 23], including but not limited to their perceived lack and demonstration of graduate attributes [19]. Considering the reality of managing patients in a setting with real consequences for actions [24] and the preclinical years being marked by mostly didactic lectures and in-class practical work [25], it should come as no surprise that academics from all five departments within the SoHRS (see Table 1) reported difficulties with the aforementioned transition of their students. This transition could be challenged due to the limited attention given to the scaffolded and practical development of GA within a decolonized, inclusive and digitalized space throughout the preclinical study years. Within the SoHRS at the UFS, the need was identified that a curriculum renewal process should be undertaken to deliberately focus on GA development, framed against the current higher education milieu (see Fig. 1). This article aims to share the theoretical underpinnings and resulting conceptualization of a preclinical skills training curriculum for healthcare students in the SoHRS.

## Methodology

This study used a multimethod research design to first thematically identify and map essential GA for a skills-based healthcare curriculum through a literature review and a consensus-seeking process. Second, quantitative and qualitative data were utilized to report on student perceptions after their initial implementation in the first year of study. Ethical approval was obtained by the General/Human Research Ethics Committee (UFS-HSD2021/0921/21) of the UFS before the study commenced.

All SoHRS staff (Table 1) were informed via email of the research aim process and invited to provide input and feedback to identify the possible curriculum content of a skills-based curriculum for SoHRS students. Participation was voluntary. Participants were guided by the key factors and principles encapsulated in the conceptual framework (see Fig. 1). As the study commenced while COVID-19 regulations were still in place, an electronic approach to sending and receiving feedback was deemed most suitable. The thematic analysis of the provided feedback allowed for the creation of the curriculum outline. The iterative nature of the process enabled continuous feedback and ensured amendments were made in line with the provided feedback. The document was circulated until consensus, indicated by no new additions or amendments suggested, was reached on the proposed curriculum outline. Upon final approval, the curriculum outcomes and teaching and learning activities were developed, and the first-year module was implemented in 2022.

Following the implementation of the first-year module, all first-year students enrolled in the module were informed of the study by means of an information leaflet through their institutional emails and invited to participate in the voluntary annual institutional module feedback survey, consisting of closed- and open-ended questions. Closed-ended questions related to module quality and assessment, support provided, use of technological elements, student-lecturer relationship and ownership of learning. Open-ended questions elicited narrative accounts of module experience and suggestions proposed by participants. The survey was made available through the institutional learning management system at the end of the academic year to all first-year students participating in the new skills development module (*N* = 135).

## Results and discussion

Within this section, a discussion of the curriculum outline, with the relevant results, will first be included. Following this, the results and discussion describing student perceptions after the initial implementation in the first year of study will be presented.

### Literature review and curriculum outline development

A literature search, based on the conceptual framework (see Fig. 1), was performed to map the GA expected on an institutional and faculty level through the exploration of the UFS value rubrics and the Faculty of Health Science’s interpretation thereof. In addition, the GA expected by the Health Professions Council of South Africa of students graduating from the SoHRS was also ensured to be contained within the curriculum outline. The literature review further included an analysis of existing curricula in the various programmes in the SoHRS to identify strengths and gaps with regard to GA development.

The literature review and analysis of existing curricula were presented to all departments in the SoHRS as a mindmap during the initial round of consensus seeking. All SoHRS departmental staff (N=36) participated in the consensus-seeking process through various rounds of feedback, incorporated into the original mindmap, with redistribution to all departments after each round. When no further input was received from any of the departments, final consensus was declared with implied approval of the skills training curriculum outline.

**Fig. 2:**
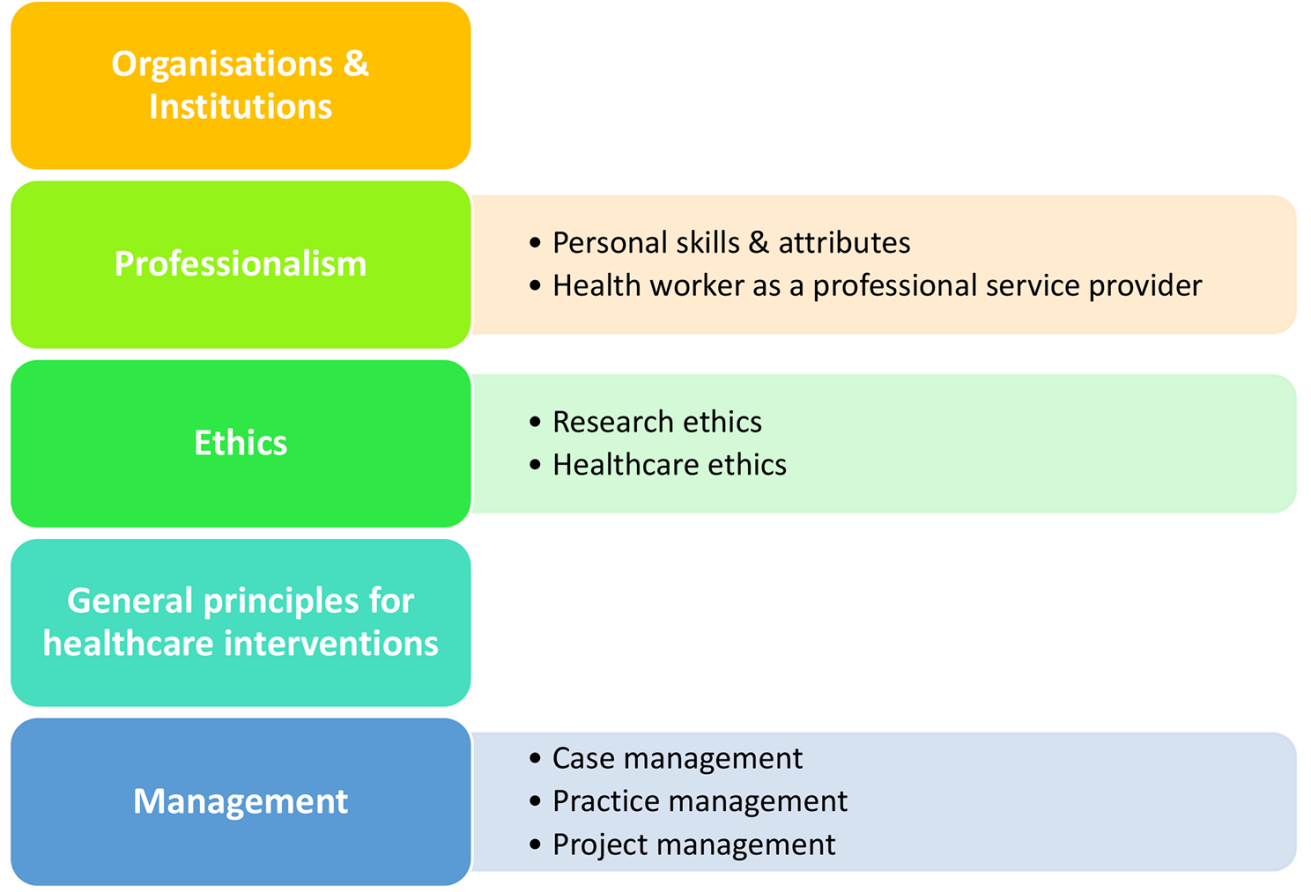
Curriculum themes and subthemes identified during the consensus-seeking phase

Five curriculum themes, namely, Ethics, Professionalism, General principles for interventions, Organizations and Institutions as well as Management, emerged with a total of seven subthemes (see Fig. 2).

The curriculum outline, as presented in Fig. 2, and how it is influenced by the conceptual framework elements from Fig. 1 are discussed in the next section.

### Curriculum themes and content

According to participant feedback during the consensus-seeking process, an introduction to healthcare Organizations and institutions, inclusive of the structure of healthcare within South Africa, was deemed an appropriate place to commence healthcare-related discussions within this curriculum. Attention to the role of accrediting bodies and other professional associations was indicated as an essential preparatory step for students to manage themselves as professionals, not only upon graduation but also as students and in their interaction with patients in various healthcare settings. Supporting the urgency to revise healthcare education toward being more contextually orientated is the imminent implementation of National Health Insurance (NHI) in South Africa. If the impact and role of such an insurance system is not discussed, graduates might struggle to find their place within the ever-changing healthcare environment.

The importance of Professionalism in a variety of situations was explicitly highlighted by participants throughout the consensus-seeking process. As illustrated in Fig. 2, two subthemes emerged from the theme of professionalism, namely, *Personal skills and attributes* and the *Healthcare worker as a professional service provider*. Elements related to professionalism as a healthcare worker and service provider should be included as soon as the first year of study for students to be clear on the code of conduct and expected healthcare professional behavior both during their studies and in healthcare practice. The clinical environment is complex and requires interactions with diverse patients, as well as students and qualified practitioners from varied professions [26]. Although students engage in these situations upon entering the clinical area, there is limited exposure to such interprofessional scenarios in the preclinical years [26], further fuelling student anxiety and uncertainty. As GA development rests heavily on interprofessional and multicultural interactions, a unified approach to embedding focused GA training within the SoHRS through an interprofessional model, including students from all departments within the SoHRS, was eminent, especially during the preclinical years. This might support students to better transition to the clinical years, with reduced anxiety and uncertainty regarding interprofessional cooperation. The essential skills of problem solving, critical thinking, academic reading, multicultural engagement, effective communication and teamwork are already expected to be mastered upon entering the clinical arena [8, 19] and should therefore be developed throughout the preclinical years.

The theme of General principles for healthcare interventions relates to aspects such as healthcare promotion and prevention as well as fostering positive patient interactions across various platforms. With globalization and the onset of the Fourth (4IR) and subsequently Fifth (5IR) Industrial Revolutions, an essential new generation of GA is also emerging. These attributes are related to sustainability, engagement with technology, and intercultural and international skills [7, 14]. The vast amount of readily available information and the increasing speed at which knowledge and technology are produced additionally necessitate the inclusion of attributes relating to critical consumerism and responsible technology use. Looking into the near future, the 5IR, considered the trust revolution, is speculated to again place increased focus on the human factor. Although the unique attributes expected from healthcare professionals might not change dramatically, a clear shift toward sustainable healthcare within a contextualized environment is unavoidable [27].

The importance of a variety of communication skills needed from a healthcare professional was highlighted through communication viewed as a cross-cutting GA aligned with not only the General principles for healthcare intervention theme but also both the *subthemes of Personal skills and attributes* (Professionalism theme) and *Healthcare ethics* (Ethics theme). Therefore, relating to more than only patient and interprofessional communication skills is viewed as important to develop in healthcare students. It has been well documented that effective communication in a healthcare setting improves patient safety, satisfaction, and compliance, in turn resulting in improved job satisfaction on the part of healthcare professionals [10]. It is thus not surprising that the development of communication skills has received much attention in the healthcare education literature [10]. To a lesser extent, other attributes, such as empathy, breaking bad news and interprofessional encounters, have also received attention [10]. We propose the scaffolding of communication skills throughout such a skills-based curriculum to assist students in moving from basic professional communication and active listening skills toward difficult conversations where empathy and cultural sensitivity are needed, culminating in research- and business-based communication in the third and fourth years, respectively.

The results from the consensus-seeking process indicated that the subthemes *Research ethics*, *Practice management*, *Project management* and, to a small extent, *Case management* be included in the third and fourth year of study, under the umbrella term Management. Irrespective of their working environment, it is vital for students to develop basic management skills to manage themselves and their patients within any healthcare environment. The addition of *research ethics* not only speaks to the mandatory accrediting bodies’ requirements for all professional degree programmes in the SoHRS to complete a research project, basic research ethics is also vital for day-to-day evidence-based practice. Participants also viewed the teaching of Management to shift from the previously more didactic teaching toward a more practical application of the theory within various environments - simulated or real-life.

Participants unanimously agreed that the curriculum be divided into four modules, presented as one module per year over the four study years. Through consideration of cognitive load theory, participants opted for a scaffolded and phased implementation of the curriculum with modules progressing in complexity from novice to expert through years one to four of the curriculum. Identification of the optimal positioning of the various skills and attributes proposed throughout the four years of study was essential to ensure that the content taught aligned with the experience level of the students. In addition to the experience level of students throughout the four years of study, it was also important to position certain skills within the preclinical years to better prepare students for the transition to the clinical years. Following this process, the first-year module was ready for implementation in 2022, and the results from the initial implementation are shared in the next section.

### Perspectives from a first implementation

**Fig. 3:**
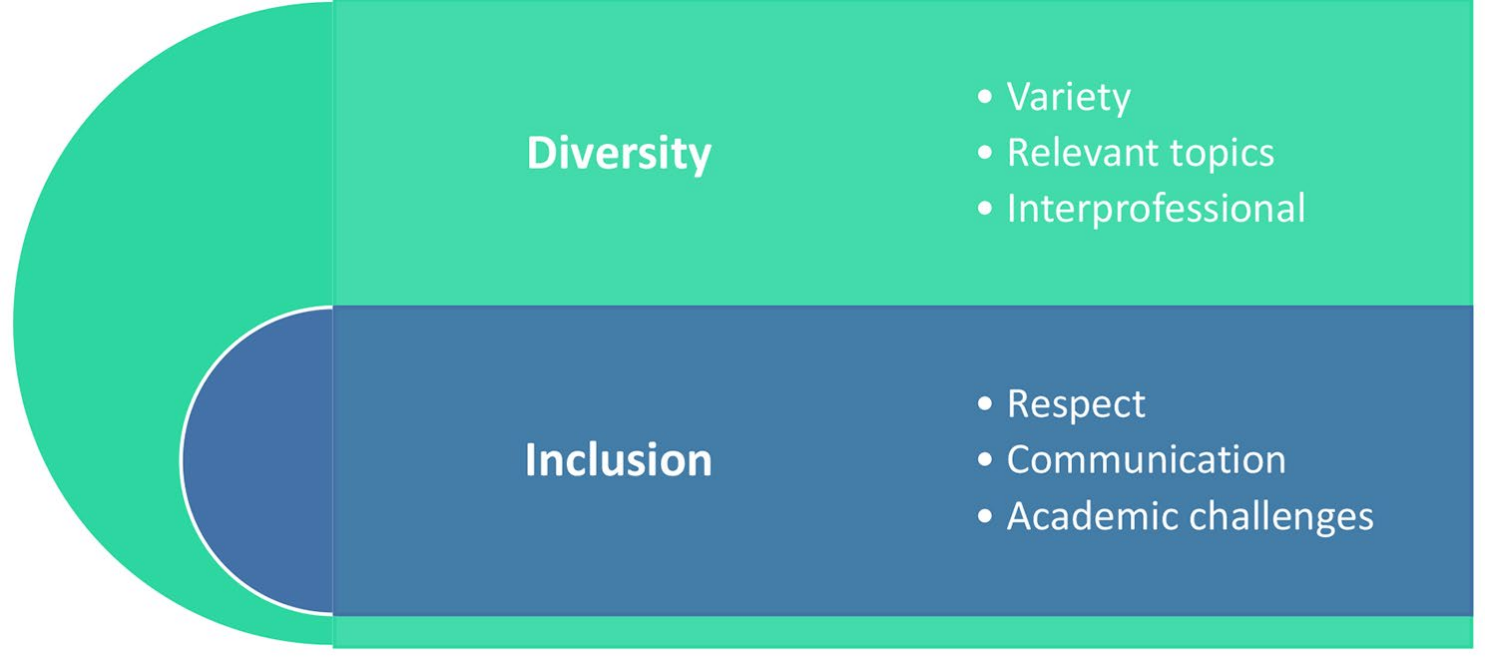
Qualitative data summary from first implementation in the first year of study

A total of 30.3% (*n* = 41; *N* = 135) of first-year students participating in the new curricular space completed the annual institutional module feedback survey online, with 87.2% (*n* = 34) of students indicating that they had a positive learning experience. Qualitative data revealed two overarching themes, namely Diversity and Inclusion, with their respective subthemes, as included in Fig. 3.

Students indicated that they “*liked that it [module] wasn’t just about one topic*” as it taught them that “*there is more to being a healthcare professional that just treating people*”. Allowing a space where students could make connections and find meaning in the content taught aimed to promote deep learning (Butcher, Davies& Highton, 2006). Although quantitative data indicated that only 58.5% of students felt they developed at least one GA as defined by the UFS, one student’s comment highlights the impact of early engagement with the implemented module, possibly spanning beyond the survey definition of GA,

*We learned about things that nobody really tells you to do, it is just expected from you in your immediate workplace*.

Students enjoyed the practical aspect of the module as they were allowed to “*apply what we have learned*.” An interactive approach to GA development has been reported to yield positive results [8], and together with results from this study, the need for a shift from theoretical to more practical GA development is highlighted. Furthermore, engaging in a community-based project hosted within the module not only facilitated the contextualized application of learned skills but was also reported to be “*quite eye-opening*” and allowed students “*to get a better perspective with what communities struggle with*”. As reported by Page et al. [7], working toward a decolonized curriculum should include elements related to intercultural education, as this is fundamental for student learning and personal growth, especially considering areas with diverse populations and globalization. Therefore, sensitizing students to aspects related to cultural sensitivity and diversity, human rights, social justice and social and civic responsibility not only in a classroom setting but also within the community was deemed essential.

Of concern is the reported limited ability of entry-level healthcare professionals to optimally function as an interprofessional team [28]. Integrating and expanding on interprofessional engagement [29] through the inclusion of external stakeholders be it in or outside of healthcare, as well as peers in deliberately created interprofessional module groups, is therefore vital. The importance of early engagement with all relevant stakeholders within the healthcare system [30] together to create an interprofessional curriculum resulted in the recruitment of a variety of presenters that students valued. Interprofessional group work also formed a large part of the first-year module. As expected, some students enjoyed working and learning with their group as it created a “*trusting environment*”, with some students commenting on the negative aspect of groupwork where “*some students did nothing to contribute to an assignment*”. Groupwork has the power to enhance a sense of community among students [21], and the application thereof should be reconsidered rather than removed within healthcare curricula. The authentic engagement with both experts and peers in this module allowed students to be exposed to varied perspectives on topical and social issues from the viewpoint of a healthcare student and future healthcare professional.

To align with healthcare and educational transformation, it is advised that educational encounters are experiential and structured within a self-directed framework [8, 31] to enhance personal and professional development. The multimodal approach to teaching and learning employed in the module to encourage student motivation and active learning [21, 32] was reported by students to have enhanced their learning experience (61%). Students could engage with content and each other on a variety of platforms, with individual online journal reflections allowing a space for more personal reflection, where small group discussions and in-class discussions around more topical aspects fostered knowledge coconstruction in an inclusive and “*supportive [environment]*.” Diverse module assessment strategies also aimed to ensure a fair distribution between individual versus group assessments as well as theoretically oriented assessments evenly distributed with more creative and/or reflective assessments. Strategically selecting a variety of teaching and learning opportunities and assessments may also assist in developing those abilities that students might not yet feel comfortable with [33].

The developed curriculum has the distinct aim of creating a truly inclusive community within the curricular space, thereby enhancing student engagement in the practical development of essential GA. The majority of students (82.5%) indicated that they felt the lecturer fostered engagement between all stakeholders within a space of mutual respect (82.9%). Lecturers being “*understanding and respectful*” and the deliberate assignment of diverse interprofessional module groups assisted in building “*a trusting environment and a supportive one*”. A basic human need is that of being part of a community and within a teaching and learning context, being part of such a community has been reported to enhance student engagement [21] and support the movement beyond merely passive inclusivity [5].

In creating an inclusive academic community, interpersonal communication is essential, including interactions between students and lecturers. Effective communication between all stakeholders may foster the creation of an inclusive learning community where students and lecturers can collaborate and resolve possible conflicts in a respectful, civil, and productive way [21]. One student stated that they “*appreciated the continuous communication*”, supported by 68.3% of students indicating that frequent communication with the lecturer enhanced their learning. This positive result may be due to the module communication taking on various formats ensuring that it “*was always clear what was expected*” from students, thereby decreasing any uncertainty or anxiety. Utilizing the power of constructive feedback throughout the curriculum, be it through self-, peer- or facilitator-evaluation, may have aided the development of GA, as complex aspects require time to develop [11].

Not only was lecturer-student communication cited as an essential component in positively influencing the learning experience (Barkley, 2010) but even more so communication within the broader community [5]. Communication between healthcare professionals and their patients, including the interprofessional team, is vital in ensuring an optimally functioning healthcare system. It was therefore decided to introduce first-year students to the basic aspects relating to communication, culminating in them applying their newly acquired skills within the community as part of the community-based project. Students were even able to identify their shortcomings, as stated by one student it *“…helped me see that I need to work on my verbal communication skills*” with an appreciation of the value of learning how to “*interact with different individuals of the community*”. Cohesion between content not only in the curriculum but also what is expected of students outside the curriculum is important for students to make the connection between their university and workplace learning [11]. In the final unit of the year, students took their skills development one step further and critically reflected, at the hand of literature, on their communication-related experiences within the community. Linking the concepts of multicultural communication, professional behavior, social justice, and academic writing, students have experienced the first steps in becoming critical consumers of literature, health advocates, and most importantly reflective practitioners.

The importance of personal well-being and self-management cannot be ignored as foundational concepts within a curriculum aiming to develop GA. Student support is paramount to student success and forms the foundation of the strategic goals of the UFS. Freeing up time for students by scheduling no department-specific contact sessions during the allocated weeks allowed students to ensure that their “*full attention was on [the module]*” and was experienced in a positive way, as it did not “*add more stress to [our] schedules*”. In contrast, one student indicated a feeling of being overwhelmed as “*the addition of this module made the workload unbearable at times*”, although a lack of self-management was cited by another student as a reason for falling behind at times. The perceived lack of academic support (61%) may have contributed to students feeling uncertain as to how to manage the workload. However, with only more than half of the students (53.7%) reporting that they engaged with additional resources provided, it might have negatively influenced students’ perception of the academic support provided. Considering that the overarching goal of the curriculum and module is that of GA development, academic support in the form of tutorials and additional academic activities does not directly influence the development of GA, although it remains a point of concern. As this module forces students to engage with various formats and platforms of knowledge, aiming to make digitalization part of their educational journey, additional support in terms of basic digital skills and navigation of the institutional learning management system could support students with the transition to a digitalized teaching and learning environment.

## Conclusion

Through the creation of a diverse and inclusive curricular space set within a multimodal and interprofessional environment, a positive learning experience was created for the development of GA.

Acknowledgment of the evolving nature of healthcare education in terms of GA development, decolonization and digitalization speaks to the importance of communication between all those participating in the curriculum to ensure appropriate integration of the aforementioned concepts. A practical approach to the development of GA was widely accepted by students, notably with the inclusion of various experts to engage with students in their areas of expertise. Digitalization, especially in learning and teaching, allows for global participation by less developed economies; knowledge and resource sharing has never been easier. Investigating opportunities for facilitators to join from across the country as well as abroad would further enhance the interprofessional nature and impact of this curriculum.

The conceptual framework and resulting insights have proved valuable at this South African institution to further develop and implement the first year of this curriculum and could prove valuable for other institutions in similar higher education environments. When designing an interprofessional curriculum, a shared narrative between staff members involved in the curriculum was viewed as essential in this study and provided valuable and diverse input. Future research should investigate the longer-term impact of this teaching and learning approach on the development of GA during the transition of these students into the clinical training space. Within healthcare education, we are acutely aware of the workload of our undergraduate students, and investigating the provision and best format of additional academic support should be investigated, as it may assist students in developing essential self-management skills to manage conflicting academic and personal engagements.

Due to the study being conducted at only one institution as well as the small sample size of first-year students completing the survey, the results may not be generalizable to other contexts. However, considering the universally important content identified for curricular inclusion and the initial positive results obtained, similar research is advised in a variety of contexts. The questions in the module survey might have been too general to obtain in-depth insight into the value addition of the first-year module. A recommendation could be the inclusion of reflective essays or journalling to explore students’ experiences and perceptions of the content taught as well as the impact of how it was taught.

## Data Availability

Additional datasets used and analyzed during the current study are available from the corresponding author upon reasonable request.

## Abbreviations

GA: Graduate attributes
SoHRS: School of Health and Rehabilitation Sciences
